# Enhancing clinician and patient understanding of radiology reports: a scoping review of international guidelines

**DOI:** 10.1186/s13244-020-00864-9

**Published:** 2020-05-05

**Authors:** Caitlin I. Farmer, Allison M. Bourne, Denise O’Connor, Jeffrey G. Jarvik, Rachelle Buchbinder

**Affiliations:** 1 Monash Department of Clinical Epidemiology, Cabrini Institute, 4 Drysdale St, Malvern, VIC 3144 Australia; 2 Department of Epidemiology and Preventive Medicine, School of Public Health and Preventive Medicine, Monash University, Melbourne, VIC Australia; 3 Departments of Radiology, Neurological Surgery, School of Medicine and Health Services, School of Public Health, University of Washington, Seattle, WA USA; 4 Departments of Pharmacy and Orthopaedic & Sports Medicine, School of Medicine, University of Washington, Seattle, WA USA

**Keywords:** Comprehension, Diagnostic imaging, Guidelines as topic, Radiology, Review

## Abstract

Imaging reports are the primary method of communicating diagnostic imaging findings between the radiologist and the referring clinician. Guidelines produced by professional bodies provide guidance on content and format of imaging reports, but the extent to which they consider comprehensibility for referring clinicians and their patients is unclear. The objective of this review was to determine the extent to which radiology reporting guidelines consider comprehensibility of imaging reports for referring clinicians and patients.

We performed a scoping review of English-language diagnostic imaging reporting guidelines. We searched electronic databases (OVID MEDLINE, Embase) and websites of radiological professional organisations to identify guidelines. The extent to which the guidelines recommended essential report features such as technical information, content, format and language, as well as features to enhance comprehensibility, such as lay language summaries, was recorded.

Six guidelines from professional bodies representing radiologists from the USA, Canada, Australia and New Zealand, Hong Kong, the UK and Europe were identified from the search. Inconsistencies exist between guidelines in their recommendations, and they rarely consider that patients may read the report. No guideline made recommendations about the reporting of results considering the clinical context, and none recommended features preferred by patients such as lay language summaries. This review identifies an opportunity for future radiology reporting guidelines to give greater consideration to referring clinician and patient preferences.

## Key points

- Radiology reporting guidelines produced by international radiology professional bodies are focused on technical detail and structure of the report.
- Radiology reports are increasingly accessed by a wide range of healthcare clinicians with varying levels of expertise, as well as patients themselves, and it is important they understand a report as it was intended.
- International guidelines rarely consider many of the preferences for radiology reporting expressed by referring clinicians and patients which may lead to confusion and anxiety.

## Introduction

Outside of hospital settings, where co-located clinicians and radiologists are able to more easily communicate, diagnostic imaging requests and reports are the primary means by which referring clinicians and the radiologists who report imaging findings communicate with each other [1]. The report may also be read by a range of other healthcare professionals with varying levels of experience and knowledge. It is therefore imperative that both the requests and reports are understood in the way they are intended in order to inform appropriate clinical decisions.

It is widely accepted that diagnostic imaging reports must provide an accurate and detailed interpretation of the imaging findings. Less clear is exactly how that message should be communicated. Radiology reports vary widely in terms of phrasing, length and clarity [2], and there is growing evidence that referring clinicians and patients interpret ambiguous phrasing in radiology reports with more concern than radiologists, increasing patient anxiety and rates of follow-up testing [3]. In a study of 15 different phrases commonly used to convey the level of diagnostic certainty, radiologists and referring clinicians only agreed on one phrase (‘diagnostic of’) [4]. Along with diagnostic ambiguity, the use of more medical or precise terminology to describe a condition has been shown to lead to higher levels of patient anxiety, as well as perceptions of increased severity of the condition, and patient preference for more invasive treatments [5].

Professional member associations such as the American College of Radiology (ACR) [6] and the Australian and New Zealand College of Radiologists (RANZCR) [7] have guidelines regarding the content and structure of radiology reports. These guidelines aim to improve the quality and utility of imaging reports. The objective of this review was to determine the extent to which radiology reporting guidelines consider the preferences of the referring clinician and patient particularly with regard to comprehensibility of imaging reports.

## Methods

### Design

We conducted a scoping review using the methodology described by Arksey and O’Malley [8] and Levac et al. [9]. We reported our search and selection results according to the PRISMA Extension for Scoping Reviews (PRISMA-ScR) [10].

### Selection criteria

We included all guidelines for communication of diagnostic imaging results created and published by a radiology professional body or a national member organisation and available in English. Guidelines in other languages without an official English translation were excluded as adequate translation services were not available; however, Google translate was used to identify potentially relevant publications in other languages. Publications from organisations other than radiology professional bodies, experimental studies, surveys, opinion pieces, editorials, guidelines regarding interventional radiology and guidelines on radiology research were also excluded as were research papers used to inform guidelines and condition-specific guidelines, such as the Thyroid Imaging Reporting and Data System (TI-RADS) for reporting incidental thyroid nodules [11].

### Search methods for identifying guidelines

We searched all 57 available websites of national member radiology societies associated with the International Society of Radiology (http://www.isradiology.org/2017/isr/index.php). We also searched OVID MEDLINE and Embase from inception to 26 March 2019. The search strategy was developed in conjunction with an experienced librarian, and for MEDLINE, it was the following:

1. ((imag* or radiolog*) adj5 (result* or report* or record* or outcome*)).ti.
2. (Recommendation* or practice* or guideline* or guidance or standard* or protocol* or instruction* or information or method or convention).ti.
3. 1 and 2
4. Exp animals/ not humans.sh.
5. 3 not 4

The search strategy for Embase was similar except that line 4 was replaced with (exp animal/ or nonhuman/) not exp human/.

The reference lists of included guidelines and relevant articles were reviewed to identify additional guidelines. Only the most recent version of guidelines from each organisation was included.

### Screening and selection

**Fig. 1:**
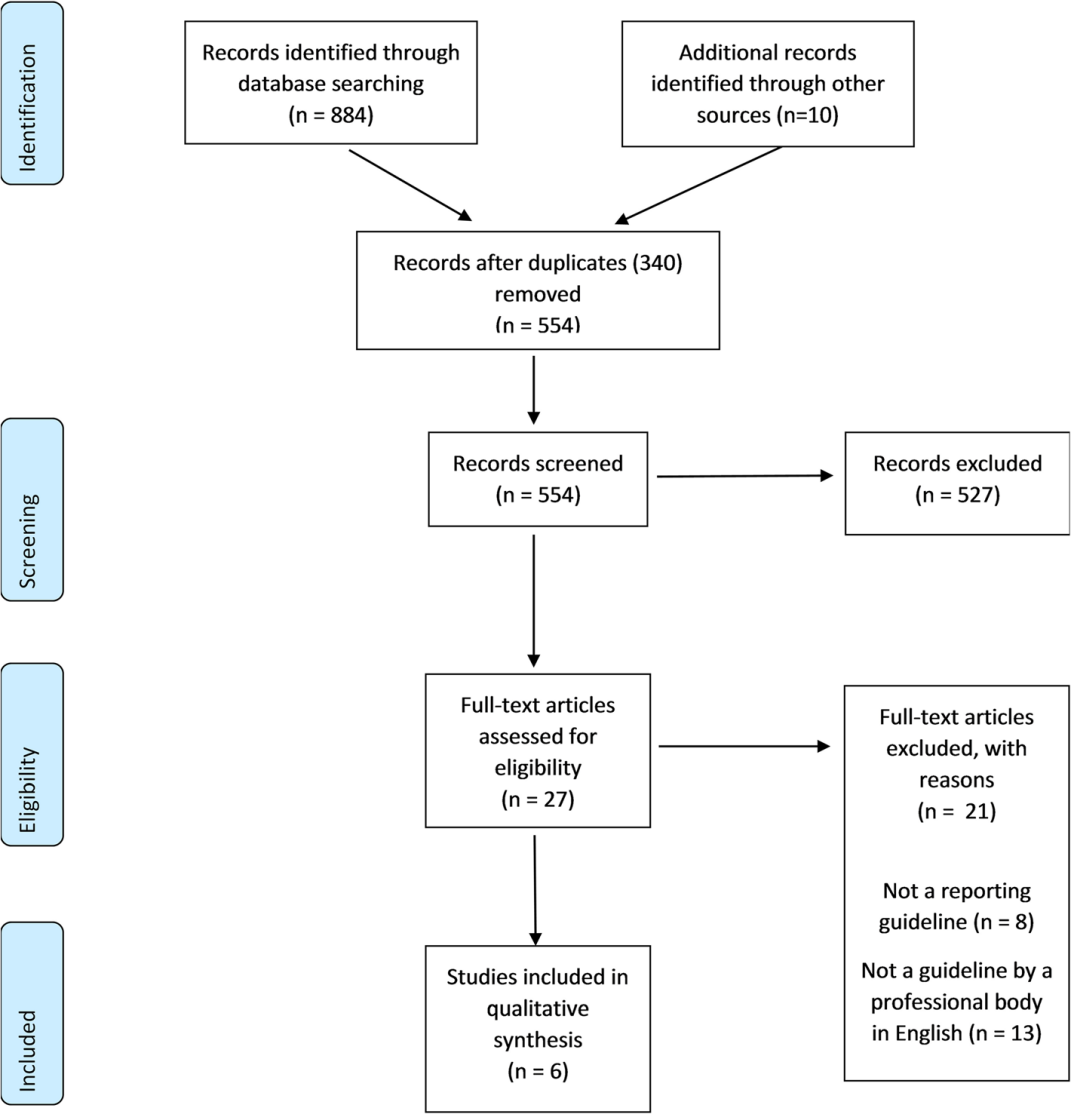
PRISMA diagram

Two review authors (C.F. and A.B.) independently screened the titles and abstracts identified by the search. Full-text reports of potentially eligible guidelines were obtained and screened independently by two review authors (C.F. and A.B.). Discrepancies were to be resolved by discussion, but there was no discordance between reviewers. A PRISMA flow chart was developed to summarise the search and selection process (Fig. 1).

### Data extraction and synthesis

A recent evidence-based guideline for the written radiology report that included a literature review, multi-disciplinary panel and public consultation [12] and which informed the RANZCR guidelines [12] was used as a template to extract recommendations for each guideline. This template can be grouped into four broad areas: technical information (patient demographics, comparison with prior studies, technique, procedural information, report status and examination quality), content (clinical information, relevant or abnormal findings, normal findings, addressing the clinical question, differential diagnosis, conclusion, recommendations for further testing or treatment and any discrepancy documentation), format (length, structured reporting, terminology for referring clinicians and patients, accuracy and ‘actionable’ reporting) and language (conveying confidence and certainty, clarity and readability).

In addition, we examined the included documents for any guidance regarding making reports more comprehensible to the clinician and/or patient, for example through suggesting lay summaries, altering or simplifying wording or provision of specific images or diagrams. Any specific advice regarding communication of findings and how this was conveyed (i.e. direct quotes) were also extracted. All findings were extracted and tabulated independently by two authors (C.F. and A.B.). Discordance was resolved by discussion and consensus.

## Results

Of the 611 potentially eligible documents we identified, 27 were included for full-text review and six satisfied our eligibility criteria [6, 7, 13–16] (Fig. 1). A further four documents were identified on the websites of the Spanish Society of Medical Radiology, Italian Society of Radiology, Latvian Association of Radiologists and German Roentgen Society; however, these were excluded as no English language versions of these documents were available. The included documents comprised the guidelines from RANZCR [7], the UK Royal College of Radiologists (RCR) [13], the ACR [6], the Canadian Association of Radiologists (CAR) [16], the Hong Kong College of Radiologists (HKCR) [14] and the European Society of Radiology (ESR) [15]. Three guidelines were published in the last 2 years [7, 13, 14]. The ACR guidelines were revised most recently in 2014 and the ESR guidelines were published in 2011 and the CAR guidelines in 2010.

### Guideline development process

Explanation regarding how each guideline was developed was variable (Table 1). All except the HKCR guidelines attributed development to committees, generally professional standards groups, and some named the individuals involved. The RANZCR guidelines state ‘development was initially achieved by a multi-disciplinary team using a transparent and documented process of integration of evidence with expert opinion’. This is presumed to refer to an initial literature review [17] which informed a project managed by RANZCR resulting in an evidence-based guideline for the written radiology report [12]. The most recent RANZCR guidelines, included in this review [7], were preceded by an online survey of clinical radiologists with updates to the previous version clearly identified [18]. This was the only guideline to include a consumer as part of the development process and one of two (the other being the ACR) to include non-radiologist stakeholders in guideline development.

**Table 1:** Provenance of included guidelines

	Author credited	Who wrote the guidelines?	How did they decide what to include?	Non-radiologists/other stakeholders included?
Royal Australian and New Zealand College of Radiologists	Copyright—College	Quality Radiology Written Reports Working Group (members named) Safety, Quality and Standards Committee and the Faculty of Clinical Radiology Council (not named)	Development described in background, ‘based on a literature review’	Yes—in survey and original group
Royal College of Radiologists (United Kingdom)	Copyright—College Principal author named in forward	Clinical Radiology Professional Support and standards Board Clinical Radiology Faculty Board Medical Director Professional Practice Clinical Radiology (journal) Not all members named	Unclear	Nil evidence
American College of Radiology (USA)	ACR	Reviewing committee, Practice Parameters committee, Comments reconciliation committee (members named)	ACR guidelines on developing practice parameters—process clearly stated on website	Not clearly stated However, the Committee on practice parameters (general, small and rural practice) was consulted and includes multiple non-radiologists. Nil evidence of consumer inclusion
Canadian Association of Radiologists	College, also acknowledges ACR guidelines	Four authors named	Based on ACR guidelines, otherwise unclear	Nil evidence
European Society of Radiology	ESR	ESR subcommittee on Audit and standards (members named) and approved by executive council	Unclear	Nil evidence
Hong Kong College of Radiologists	None. Acknowledges RCR 2012 guidelines and ESR guidelines	Unclear	Unclear	Nil evidence

### Items covered across the guidelines

All guidelines included basic suggestions regarding items to include in a radiology report (Table 2), and most with the exception of the HKCR suggested the following reporting sequence: clinical information, relevant findings, addressing the clinical question, providing differential diagnoses where required, and conclusion. The RANZCR guidelines included all the recommendations for written radiology reports regarding technical information and content based upon the template which was developed for this purpose. The HKCR guidelines were most limited, with a focus on timeliness and communication methods for radiological findings rather than report content. All other guidelines recommended the inclusion of technical information such as technique, examination quality, comparison with prior studies and procedural details. The same five guidelines recommended information regarding clinical history, relevant or abnormal findings, addressing the clinical question, differential diagnosis and conclusion. All six guidelines discussed recommendations for further testing or treatment, but only three recommended reporting normal findings [7, 13, 15]. Documentation of any discrepancies between an initial and final report was recommended in three guidelines [6, 7, 16].

**Table 2:** Comparison of advice to radiologists regarding items to include in imaging findings

	Royal Australian and New Zealand College of Radiologists	Royal College of Radiologists (UK)	American College of Radiology	Canadian Association of Radiologists	European Society of Radiology	Hong Kong College of Radiologists
Technical information						
Patient demographics	✓	✓	✓	✓		
Report status	✓	✓	✓	✓		
Comparison with prior studies	✓	✓	✓	✓	✓	
Technique	✓	✓	✓	✓	✓	
Procedural description	✓	✓	✓	✓	✓	
Examination quality	✓	✓	✓	✓	✓	
Content						
History/clinical information	✓	✓	✓	✓	✓	
Relevant or abnormal findings	✓	✓	✓	✓	✓	
Normal findings	✓	✓			✓	
Addressing the clinical question	✓	✓	✓	✓	✓	
Differential diagnosis	✓	✓	✓	✓	✓	
Conclusion	✓	✓	✓	✓	✓	
Testing or treatment recommendations	✓	✓	✓	✓	✓	✓
Discrepancy documentation	✓		✓	✓		

### Target audience for report

All guidelines emphasise the need for accuracy, consider the referring physician the main audience and provide structure around language required. All encourage ‘actionable’ reporting, where radiology images are transformed into reports that assist patient care and influence outcome [19]. Four guidelines recommend the use of terminology should consider the referring clinician’s background and not be overly technical [6, 7, 13, 15]. Only two guidelines explicitly note patients and/or their carers may view results and recommend this be considered in reporting [7, 13].

### Discussions of clinical certainty

Three guidelines [7, 13, 16] discuss reporting with confidence or certainty. All guidelines make some reference to the report being ‘clear’ [13–15] or advocate for brevity [7], and most suggest that the final report should be carefully reviewed to ensure there are no ‘confusing or conflicting statements’ [16]. Two guidelines make some reference to the readability of the report [7, 15], although the ESR guidelines merely suggest avoiding ‘long descriptions of limited use to the referrer’. The RANZCR guidelines make direct reference to readability, which appears to be used interchangeably with the notion of clarity. However, this is not clearly defined in the guidelines or the papers on which the guidelines were based [12, 17, 18]. One guideline suggests including ‘a conclusion or summary of the key findings in the clinical context’ [13]. No guidelines in this review considered provision of lay summaries aimed at patients (Table 3).

**Table 3:** Comparison of advice to radiologists regarding communication of imaging findings

	Royal Australian and New Zealand College of Radiologists	Royal College of Radiologists (UK)	American College of Radiology	Canadian College of Radiologists	European Society of Radiology	Hong Kong College of Radiology
Purpose of the report	Not discussed	‘The purpose of an imaging report is to provide an accurate interpretation of images in a format that will prompt appropriate care for the patient’	‘The final report is the definitive documentation of the results of an imaging examination or procedure’	‘The effective transmission of imaging information from the radiologist to the referring physician constitutes the main purpose of the report’	‘The written radiology report is the most important means of communication between the radiologist and referring medical doctor’	‘..the written radiology report constitutes the legal record of the radiology investigation or procedure’
Length	‘Reports should be as concise as possible while still conveying the information required….’	‘Where there is a need for a long descriptive report, it should conclude with a short summary of key findings and their interpretation…’	Not discussed	‘The ideal radiology report is ….concise’	‘A balance needs to be struck between a clear description of the positive and negative findings and the concentration of the reader’	‘[The responsibility of the radiologist is to] '…ensure that the reports are….precise’
Templated or structured reporting	‘Standardised examination/disease process-specific templates should be developed where they are likely to improve the quality of communication…’	Not discussed	‘Standardized computer-generated reports should be designed to satisfy the above criteria’	‘Standardized computer-generated template reports (or other structured report formats) that satisfy the above criteria are considered to conform to these standards’	‘[Structured reporting]…is more time efficient than dictation….has also been suggested to improve communication of radiology results…’	Not discussed
Terminology—referring clinician	‘…should use terminology with widely understood and commonly agreed meaning among health care practitioners.’	‘The wording of the report is likely to differ when it is written to a general practitioner who may be unfamiliar with a relatively rare condition, compared with a specialist in that particular field’	Not discussed	Not discussed	‘The wording of the report should take into account the expected level of knowledge and expertise of the referrer’	Not discussed
Terminology—patients	‘…bear in mind that a consumer may also read the report’	‘Patients now have access to medical correspondence about them... This should be borne in mind in the wording and style of the report’	Not discussed	Not discussed	Not discussed	Not discussed
Accuracy	‘Relevant imaging findings should be characterised as specifically as possible’	‘….[the reporter] should be aware of the likely accuracy of the examination in that particular patient related to the published accuracy of the technique and its applicability to this particular examination…..’	‘The report should use appropriate anatomic, pathologic, and radiologic terminology to describe the findings’	‘Use precise anatomical, radiological and pathological terminology to describe the findings accurately’	‘[The findings] section should include a targeted, systematic and comprehensive description of all abnormalities….the description should be specific…’	‘It is …vital that the information contained within this record is accurate…’
‘Actionable reporting’	‘Specific clinical questions asked by the referrer must be addressed….’	‘A radiology report should be actionable and prompt appropriate care for the patient’	‘A specific diagnosis should be given when possible….a differential diagnosis should be rendered when appropriate…’	‘Give a precise diagnosis whenever possible… give a differential diagnosis when appropriate’	‘The report may give suggestions for further action to be taken….these suggestions should be carefully considered…’	[The responsibility of the radiologist is to] ‘…clearly document advice on further management or action, where appropriate’
Confidence and certainty	‘[The report should] avoid vague modifiers such as “might be consistent with” and “possibly represents”’	‘The level of certainty or doubt surrounding an imaging diagnosis should be clearly indicated in the report’	Not discussed	‘Descriptive reporting that offers no opinion, or guidance for the resolution of the clinical question should generally be avoided’	Not discussed	Not discussed
Clarity	‘[The report should] use short sentences in preference to long sentences in prose reports and in the free text fields of itemised reports’	‘The written report should be clear, and written in a way appropriate to the referrer's expected level of familiarity with the imaging abnormalities detected…..’	‘Use of abbreviations or acronyms should be limited to avoid ambiguity’	‘The report should be clear and concise’	‘Observations should be as precise as possible, avoiding loose terms…’	[The responsibility of the radiologist is to] ‘…ensure reports are …clear and precise’
Readability	‘Clinical radiologists should review, edit and sign/authorise their own reports….to improve accuracy, clarity, readability, succinctness and logical order or examination findings, and their interpretation’	Not discussed	Not discussed	Not discussed	‘Long descriptions of limited use to the referrer should be avoided’	Not discussed

## Discussion

Based upon the six English language guidelines we were able to access, most tend to focus on structure of the report and technical information. Three guidelines encourage radiologists to consider the specialty and background of the referring clinician, while two acknowledge that patients may access their reports. Recommendations regarding format and language are inconsistent between guidelines. Only one guideline suggests the inclusion of clinical context [13], and no guideline recommends inclusion of a lay summary for patients. All guidelines suggest providing recommendations for further testing or treatment where appropriate.

Imaging reports are powerful. Radiologist recommendations in the report influence whether patients are referred for further testing [20], while report reminders regarding evidence-based practice can result in changes in prescribing [21, 22] and imaging referrals [23]. Despite this, only the RCR and RANZCR guidelines acknowledge that the way the imaging report is presented can impact patient management. The RCR guidelines state ‘the purpose of a radiology report is to provide an accurate interpretation of images in a format that will prompt appropriate care for the patient’ [13], and the RANZCR guidelines acknowledge the radiology report ‘…has an important impact on decisions about further investigation and management. Its form and content can be influential in reducing harm to patients…’ [12]. With increasing accessibility of sensitive imaging modalities such as CT and MRI, the likelihood of identifying unexpected or incidental anatomical abnormalities has increased. Such incidental findings can be more common than the condition for which the imaging is being performed to detect [24]. While detailed reporting can enable the clinician to match radiological features to the patient’s symptoms, with increasing detection of low-risk incidental findings comes a need to ensure imaging reports convey findings in a manner that enables accurate clinical decision-making and minimises potential patient harms from overdetection.

Structured reporting was discussed by four of the guidelines in this review [6, 7, 15, 16], although in one it is discussed as a potential future development [15] and in two it was suggested that any structured report should include the information included in that guideline [6, 16]. Only the RANZCR guidelines considered structured reporting in any depth, stating ‘Standardised….templates should be developed where they are likely to improve the quality of communication, and in particular, to meet the content requirements of specific referrer groups’. This ambiguity may be reflected in one Australian survey, where only 32.5% of oncologists reported regularly receiving structured reports, and 21% never received them, despite expressing a strong preference for such reports [25]. Alongside being a possible solution to radiology report interpretation issues such as error rate [26] and clarity [27] and for clinical situations such as surgical planning [28], clinicians can extract information from structured reports more easily [29, 30] and it can improve agreement between clinicians regarding the interpretation of findings [31].

Providing structured reports alone may not be enough. Primary care physicians require certainty and clinical context from radiology reports. They prefer clear indications of the meaning of radiology terminology, likelihood of disease and clinical relevance of findings [32], including the normal sizes of anatomical structures [33]. Three guidelines in this review suggest using terminology that is widely understood or appropriate to the background of the referring clinician, and three discuss conveying confidence and certainty, but only one recommends clear statements regarding the likelihood of disease. Clinicians are more likely than radiologists to prefer the inclusion of negative findings [34], something considered only by the RANZCR guidelines. Although all guidelines recommended giving treatment or management suggestions in the report, only primary care physicians appreciate this information [35], and when suggestions are given, most clinicians feel obliged to follow recommendations [36].

Although patients desire access to their report [37], and are increasingly receiving it through their electronic medical records, only two guidelines consider the patient, and only to state that the reporting radiologist should consider that the patient may read the report. Methods to reduce patient distress and anxiety that have been explored include rewording imaging reports to use simpler and more neutral language [38], including patient-oriented explanations of complex medical terms along with diagrams [39, 40], and lay language summaries [41]. Insertion of benchmark epidemiological data providing information similar to normal ranges for laboratory tests has also been proposed and investigated [42, 43]. No guidelines in this review suggested aiming reports at a specific literacy level, or using reporting techniques preferred by patients undergoing imaging.

This paper has a number of strengths and limitations. The broad search strategy ensured that all professional association websites were searched and that any published guidelines were identified. Our study is limited to guidelines produced by professional member bodies and may not represent all reporting guidelines used by radiologists. The inclusion of only English language documents may mean our results are not generalizable to guidelines in other languages.

Current radiology reporting guidelines do not reflect the preferences expressed by referring clinicians and patients for radiology report comprehensibility. In practice, while radiology reports that conform with reporting guidelines are likely to be technically accurate, these may not be understood by the referring clinician and patient in the way intended. This may lead to anxiety and potentially unnecessary tests or treatment. Given the role radiology reports play in clinical decision-making, professional radiology member organisations have a responsibility to ensure that their guidance to members considers the clarity of radiology reports for referrers and patients. We recommend that future guideline development panels include end-users including consumers to ensure the requirements of these groups are met. This review identifies how guidelines can encourage radiologists to optimise the diagnostic imaging report to best meet the needs of referring clinicians and patients.

## Data Availability

All guidelines used in this review are publicly available on the website of the relevant radiology college. Other data and materials will be shared by the authors upon reasonable request.

## Abbreviations

ACR: American College of Radiology
CAR: Canadian Association of Radiologists
CT: Computed tomography
ESR: European Society of Radiology
HKCR: Hong Kong College of Radiology
MRI: Magnetic resonance imaging
PRISMA: Preferred Reporting Items for Systematic Reviews and Meta-Analyses
PRISMA-ScR: Preferred Reporting Items for Systematic Reviews and Meta-Analyses—Scoping Review
RANZCR: Royal Australian and New Zealand College of Radiologists
RCR: Royal College of Radiologists
TI-RADS: Thyroid Imaging Reporting and Data System
